# Initiation of Pulmonary Fibrosis after Silica Inhalation in Rats is linked with Dysfunctional Shelterin Complex and DNA Damage Response

**DOI:** 10.1038/s41598-018-36712-6

**Published:** 2019-01-24

**Authors:** Mohammad Shoeb, Gul M. Mustafa, Pius Joseph, Christina Umbright, Vamsi Kodali, Katherine A. Roach, Terence Meighan, Jenny R. Roberts, Aaron Erdely, James M. Antonini

**Affiliations:** Centers for Disease Control and Prevention, Health Effects Laboratory Division, National Institute for Occupational Safety and Health, Morgantown, WV 26505 USA

## Abstract

Occupational exposure to silica has been observed to cause pulmonary fibrosis and lung cancer through complex mechanisms. Telomeres, the nucleoprotein structures with repetitive (TTAGGG) sequences at the end of chromosomes, are a molecular “clock of life”, and alterations are associated with chronic disease. The shelterin complex (POT1, TRF1, TRF2, Tin2, Rap1, and POT1 and TPP1) plays an important role in maintaining telomere length and integrity, and any alteration in telomeres may activate DNA damage response (DDR) machinery resulting in telomere attrition. The goal of this study was to assess the effect of silica exposure on the regulation of the shelterin complex in an animal model. Male Fisher 344 rats were exposed by inhalation to Min-U-Sil 5 silica for 3, 6, or 12 wk at a concentration of 15 mg/m^3^ for 6 hr/d for 5 consecutive d/wk. Expression of shelterin complex genes was assessed in the lungs at 16 hr after the end of each exposure. Also, the relationship between increased DNA damage protein (γH2AX) and expression of silica-induced fibrotic marker, αSMA, was evaluated. Our findings reveal new information about the dysregulation of shelterin complex after silica inhalation in rats, and how this pathway may lead to the initiation of silica-induced pulmonary fibrosis.

## Introduction

Approximately 2.3 million U.S. workers are exposed to respirable crystalline silica, including 100,000 who are considered at high risk^1^. Silica exposure can lead to a life-threatening, irreversible chronic lung disease referred to as silicosis that includes alveolar proteinosis, inflammation, and fibrosis. Other diseases associated with silica exposure are lung cancer, tuberculosis, and autoimmune disorders. Importantly, pulmonary fibrosis is a life-threatening lung disease characterized by difficulty breathing, frequent infections, and severe lung dysfunction with approximately 2 to 3 years survival time after diagnosis^2,3^. Occurance of this disease has been reported to be due to the repetitive epithelial cell injury and poor tissue regeneration processes, but a complete understanding of the mechanisms remains unclear^4^^.^

The initiation of pulmonary fibrosis has been shown to take place through stimulation of epithelial cells and macrophages after silica exposure, resulting in elevation of inflammatory cytokines and other mediators, promoting the phenomenon of transforming polar epithelial cells to non-polar mesenchymal cells, including epithelial mesenchymal transition (EMT)^5,6^. Furthermore, several occupational and environmental exposures can elicit damage to lung epithelial cells, leading to the increased risk of lung damage, and alteration of telomeres and its regulatory proteins^2,7–12^. Aging in humans and laboratory animals leads to shortening or dysfunction of telomeres^13^, which are protective structures at the ends of chromosomes^14,15^, and telomere shortening has been shown to occur in males and females (2:1) diagnosed with pulmonary fibrosis^16^.

Telomeres are located at the ends of chromosomes and consist of TTAGGG repeats bound by a six-protein complex known as shelterin^14,15^. The removal, disruption, knockdown, or mutation of any shelterin protein, such as protection of telomeres (POT1), tripeptidyl peptidase 1 (TPP1), TRF1-interacting protein (Tin2), telomeric repeat binding factor 1/2 (TRF1), (TRF2), and repressor activator protein 1 (Rap1), can cause genomic instability and chromosomal end-to-end fusions^17^ and results in failure to recruit telomerase and maintain the telomere^18,19^. Shelterin proteins interact with each other through binary interaction forming a high-order protein complex^15,20,21^. The TRF proteins serve as the foundation for the shelterin complex at telomeres. Both TRF1 and TRF2 are homodimers binding to the telomeric dsDNA, whereas POT1 interacts with TPP1 and binds to the telomeric ssDNA^22–24^. Both TRF proteins interact with Tin2 and stabilize the TRF1-Tin2-TRF2 interaction. Tin2 also binds to TPP1 and has been proposed to be the key component in forming shelterin complex assembly^20^. The shelterin complex is implicated in the protection of telomeres essentially through its t-loop by hiding the telomere terminus at the 3′ overhang of the dsDNA, thereby preventing dsDNA breaks^25^. Any alteration in the shelterin components can activate the canonical DNA damage response (DDR) and cause dysregulation of shelterin components^15^. The shelterin complex represses dsDNA breaks and DDR and is thought to prevent the non-homologous end joining (NHEJ) pathway which could result in chromosomal end fusions^25^.

In previous studies using telomere-deficient mice, age-related pathologies and decreased longevity have been demonstrated^26,27^. Several disease conditions, including pulmonary fibrosis, have occurred due to alteration of telomeres or their regulatory proteins and are referred to as telomere syndromes^28^. Heterodimer components of shelterin, TPP1-POT1 play an important role in telomere regulation and telomerase interaction^29^. Previous data have shown that alterations or mutations in TPP1 can result in the activation of DDR, causing uncapping and hyperextension of telomeres^30–32^. Because POT1 binds to ssDNA, depletion of POT1 can stimulate a DDR and possibly lead to telomere dysfunction, resulting in embryonic lethality, disruption of neurogenesis, and accelerated endometrial carcinogenesis^33–37^. Tin2, another crucial component of the shelterin complex, plays a central role in binding dsDNA and ssDNA shelterin components, thus forming the shelterin complex^20,38,39^. Mutations in Tin2, telomerase reverse transcriptase (TERT), telomerase RNA component (TERC), and RTEL1 genes have been reported in pulmonary fibrosis^40–42^.

TRF1-TRF2 bind to dsDNA and TRF1 and negatively regulates telomere length. Ablation of TRF1 increases telomere length in human cells, whereas overexpression reduces it^43,44^. Interestingly, knockdowns of TRF1 have been shown to induce pulmonary fibrosis and decline survival rate in mice^45^. TRF2 represses telomere t-loop cleavage, consequently playing a central role in telomere capping^46^. However, elevated expression of TRF2 have been observed in several human cancer cells and tumors and is considered as potential oncogene^47^. TRF2 induces proliferation, migration, and tube formation in endothelial cells, and its ablation inhibits these angiogenic properties. On the other hand, induction of telomere shortening has been documented in experimental TRF2 overexpression^47^. When TRF2 is compromised, robust DNA damage results as evidenced by increased accumulation of γH2AX, 53BP1, Rad17, ATM, and Mre11^48^. Rap1 is a highly conserved shelterin component united with TRF2 to prevent telomere resection and the localization of PARP1 and SLX4 at the telomeres in human and mice, thus protecting it from catastrophic telomere loss^49^. In addition, the Triple T complex, formed by the interaction of Tel2-interacting proteins 1 and 2 (TTI1) and (TTI2) with telomere maintenance 2 (TEL2), is a key regulator of phosphoinositide-3-kinase-related protein kinase (PIKKs) and is also required for several other processes, including DDR signaling, checkpoint signaling, dsDNA breakage response, and telomerase assembly in cells^50–53^. Embryonic lethality and S phase cell-cycle arrest in mouse embryonic fibroblasts have been reported in mice with homozygosity for a TEL2^−/−^ knockout allele whereas heterozygous TEL2^−/+^ were viable, fertile, and healthy^53^.

The National Institute for Occupational Safety and Health has recommended development of a more sensitive and practical approach for the analysis of silicosis^54^. Previously, we have shown telomere alteration in rat peripheral blood mononuclear cells after stainless steel welding fume exposure and in rat lung tissue after inhalation exposure to silica^12,55^. Increased expression of TERT also was observed in the lung tissue of silica-exposed rats^55^. Previously, similar silica exposure led to significantly higher pulmonary toxicity in rats at 3, 6 and 12 wk of silica inhalation compared to their respective controls. Furthermore, significantly elevated inflammatory BALF cells (AM and PMN) were also observed in silica exposed lung samples compared to controls lungs. However, silica-induced histopathological changes were not observed after 3 wk of silica exposure, which was much higher in 6 and 12 wk silica exposed animals as evident by histological analysis of the lung sections. Induction of pulmonary fibrosis activation of IL-10 and a pro-fibrotic marker MMP12 were also observed in lungs tissue of silica exposed animals^56^. Therefore, in the present study, the roles of silica-induced dysregulation of the shelterin complex and other DDR regulatory proteins as well as expression of RTEL1 were investigated. Moreover, the association of shelterin complex dysfunction between increased DNA damage protein, γH2AX, and development of silica induced fibrosis as determined by expression of a fibrotic marker, αSMA, also was evaluated. Our results, have provided new insights into the role of telomeres in the development of silica-induced pulmonary fibrosis.

## Results

### Silica inhalation affect DDR (TTI2) and RTEL1 gene

**Figure 1:**
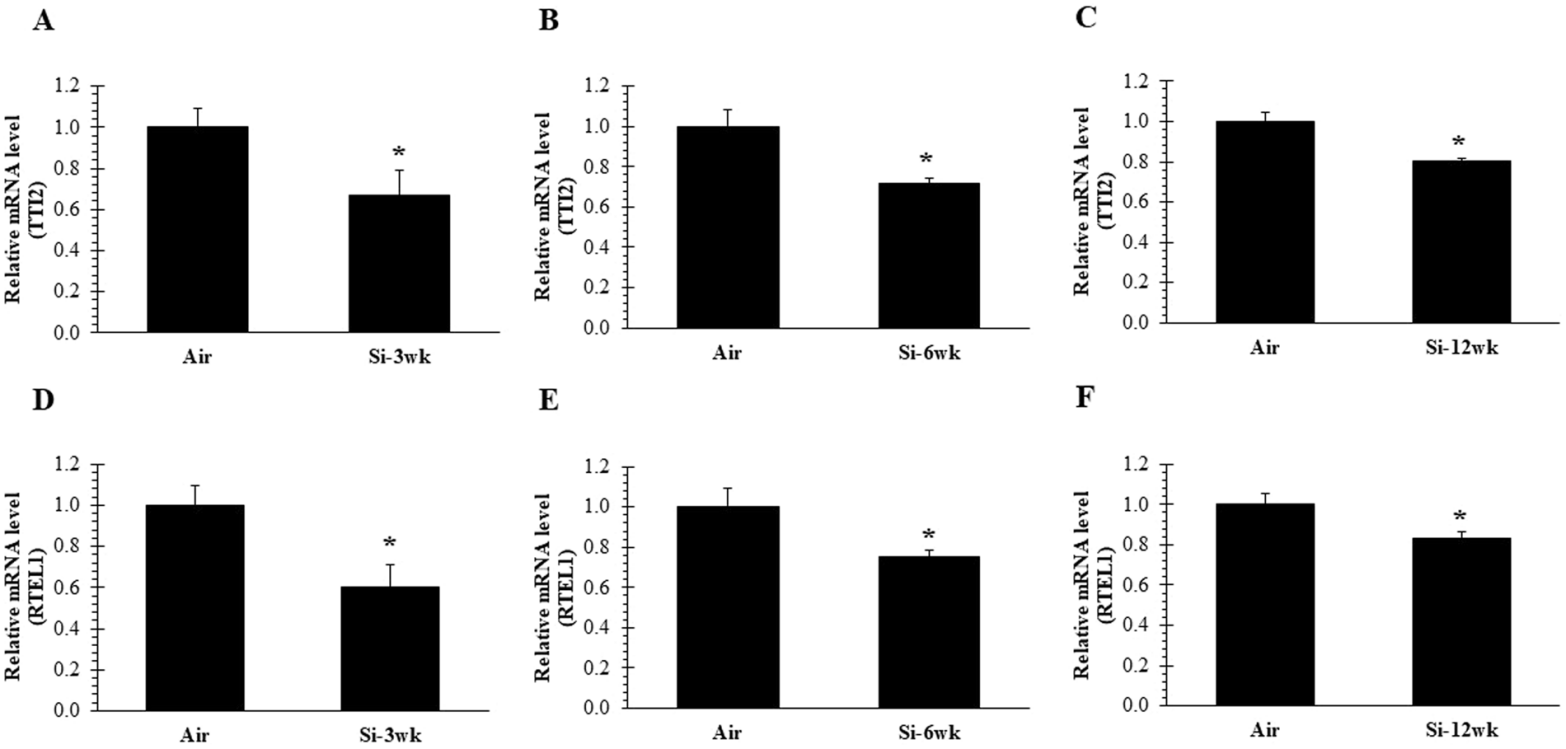
Inactivation of TTI2 and RTEL1 after 3, 6, and 12 wk of silica inhalation. (**A–C**) TTI2 and (*D–F*) RTEL1 expression in lung cDNA at 16 hr after a 3, 6, and 12 wk silica inhalation exposure (15 mg/m^3^, 6 h/d, 5 d/wk). Rats exposed to filtered air served as the controls; n = 8; values are means ± standard error; *significantly different from corresponding air control, p < 0.05.

Previously, inhalation exposure of rats to crystalline silica, resulted in minimal development of fibrosis after 12 wk silica treatment^56^. Repression of a DDR is regulated by shelterin proteins at telomeres, and plays a critical role in DNA damage^50,53^. We tested the consequences of silica exposure on TTI2, the gene involved in the maintenance of telomere length. Our data indicated a significant decrease in TTI2 expression at 16 hr after all-time points (3, 6, and 12 wk) of silica exposure as compared to air control (Fig. 1A–C). These results indicate that silica inhalation destabilizes TTI2, thus causing an activation of DDR machinery. RTEL1 is transiently associated with telomeres and is involved in the maintenance of T-Loop structure of telomeres by unwinding telomeric G4 DNA and allowing efficient telomere replication and stability. Its deletion or reduction could result in the loss of T-Loop structure^55,57^. Similar to our previous findings using an acute 5-d silica exposure regimen^55^, we observed significant decreases in RTEL1 expression in lung tissue of rats at 16 hr after 3, 6, and 12 wk of silica exposure compared to air control (Fig. 1D–F). Taken together, these results indicate that downregulation of essential DNA helicase RTEL1 may result in telomere fragility, possibly through destabilization of DDR machinery (TTI2).

### Silica inhalation enhances the γH2AX (DNA damage) and αSMA (fibrosis) in lungs

**Figure 2:**
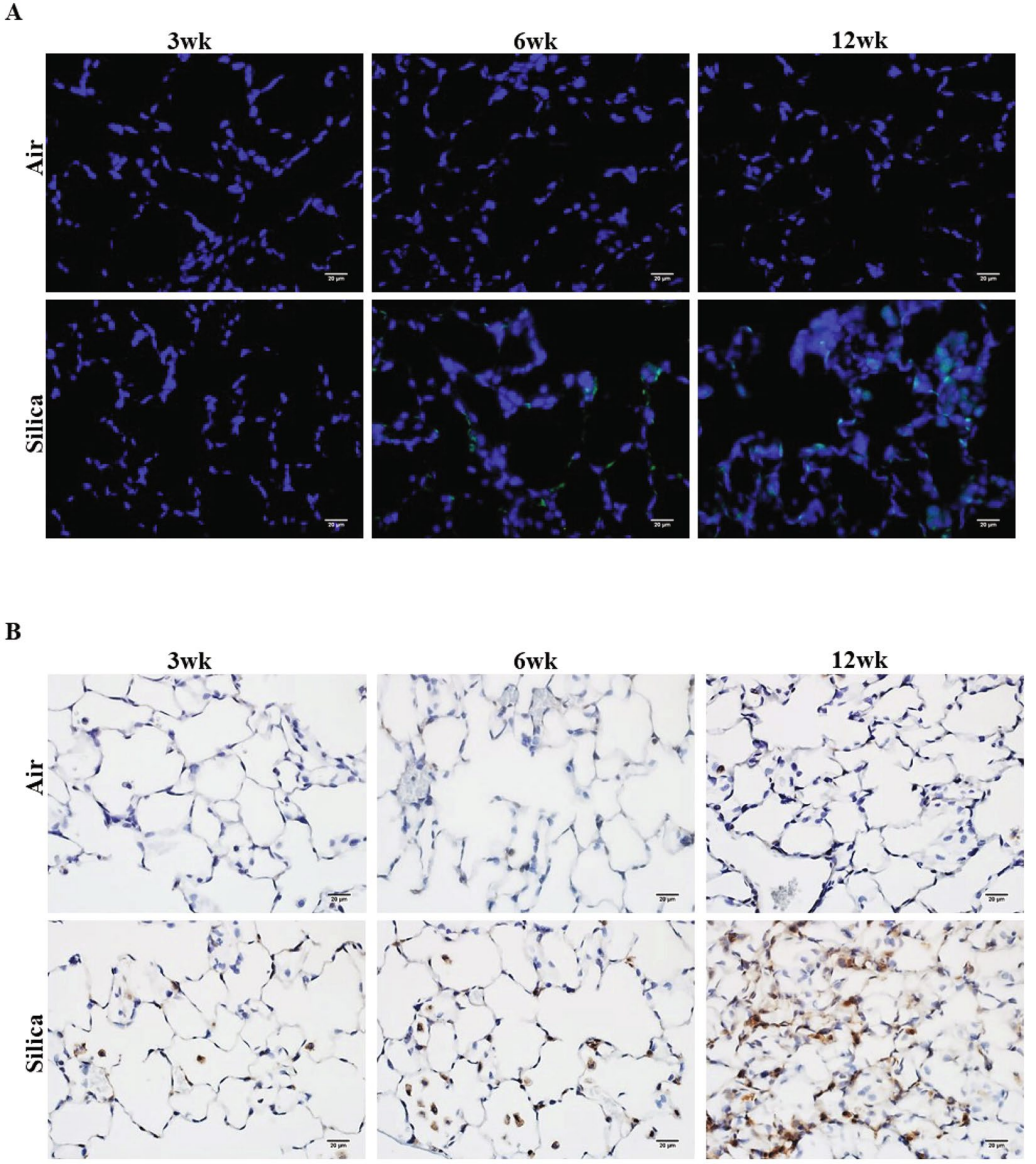
Upregulation of expression of γH2AX and αSMA after 3, 6, and 12 wk of silica inhalation. (**A**) Representative image of γH2AX positive cells (green), and (**B**) αSMA expression (brown) in lung tissue at 16 hr after a 3, 6, and 12 wk silica inhalation exposure (15 mg/m^3^, 6 h/d, 5 d/wk). The scale bar denotes 20 µm. Rats exposed to filtered air served as the controls; n = 5.

To determine the amount of DNA damage induced by silica inhalation, we respectively evaluated levels of the γH2AX (DNA damage) and αSMA (fibrosis) directly from lung sections of silica-exposed and control rats. Elevated DNA damage was observed after 3, 6, and 12 wk of silica exposure as compared to their corresponding air controls (Fig. 2A). Interestingly, DNA damage was initiated at 3 wk and was much higher at 12 wk (Fig. 2A) in the lung tissue of the silica-exposed group than in their respective air controls as observed by number of **γ**H2AX positive cells in the given field. Also, an increase in inflammation and lung injury, as evidenced by type II pneumocyte hyperplasia and initiation of lung fibrosis (mild), was observed in the same group of silica-exposed rats from a previously published study^56^. In the current investigation, we observed an increased expression of the EMT-associated marker αSMA, which has been associated with lung fibrosis^45^, in the silica-exposed groups. Increased expression of αSMA was observed at both 6 and 12 wk; however, more prominent expression of αSMA was observed after 12 wk of silica inhalation (Fig. 2B) compared to 3 and 6 wk. Collectively, from these results, we propose that upregulation of **γ**H2AX and αSMA after silica inhalation exposure can contribute to the processes of DNA damage and EMT. Together, these findings confirm the occurrence of fibrosis as a consequence of silica inhalation exposure and elevated **γ**H2AX and αSMA as evident by mild pathologic changes and pulmonary fibrosis after 12 wk of silica inhalation^56^.

### Silica inhalation-induced dysregulation of shelterin complex

**Figure 3:**
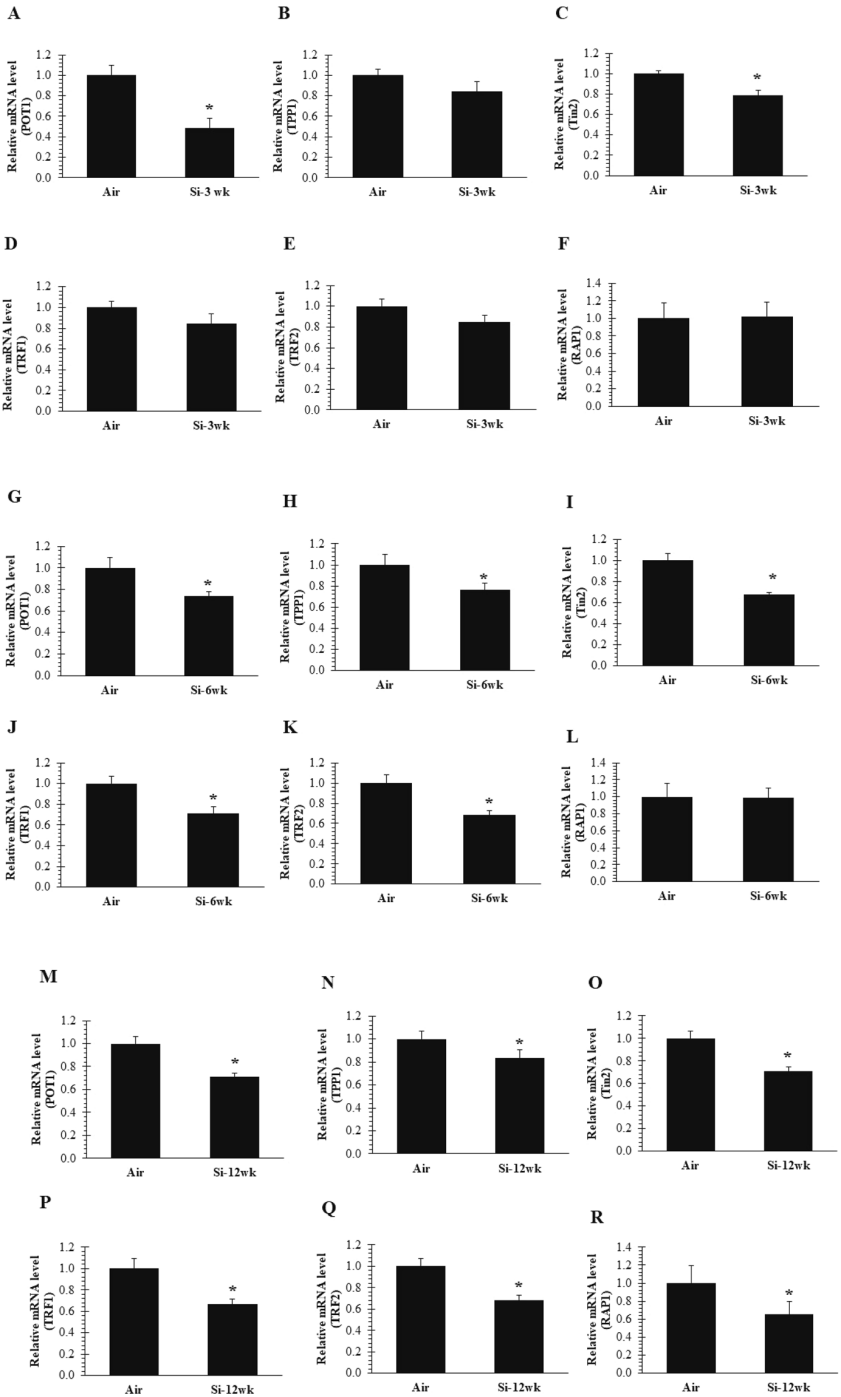
Disruption of only Tin2 of the shelterin core complex after 3 wk of silica inhalation. Inactivation of (**A**) POT1 and (**C**) Tin2 expression in lung cDNA at 16 hr after a 3-wk silica inhalation exposure (15 mg/m^3^, 6 h/d, 5 d/wk, 3 wk). (**B**) TPP1 (**D**) TRF1 (**E**) TRF2 and (**F**) RAP1 remained unchanged. All shelterin components expression (**G**) POT1 (**H**) TPP1 (**I**) Tin2 (**J**) TRF1 (**K**) TRF2 except (**L**) RAP1 were significantly downregulated in lung cDNA at 16 hr after a 6-wk silica inhalation exposure (15 mg/m^3^, 6 h/d, 5 d/wk, 6 wk). All shelterin components expression (**M**) POT1 (**N**) TPP1 (**O**) Tin2 (**P**) TRF1 (**Q**) TRF2 and (**R**) RAP1 were significantly downregulated in lung cDNA at 16 hr after a 12-wk silica inhalation exposure (15 mg/m^3^, 6 h/d, 5 d/wk, 12 wk). Rats exposed to filtered air served as the controls; n = 8; values are means ± standard error; *significantly different from corresponding air control, p < 0.05.

The shelterin complex is associated with telomeres, which protect DNA ends from a catastrophic event of dsDNA breaks that would trigger a DDR^25,38,39^. POT1 forms a heterodimer with TPP1 at 3′ G rich overhang and is required to govern chromosomal stability and maintain telomere extension by recruiting telomerase^21,22^. Here, we investigated the effect of silica inhalation exposure on the following shelterin components, POT1, TPP1, Tin2, TRF1, TRF2, and Rap1, in rat lung tissue. Gene expression analysis indicated that POT1 was significantly downregulated at all-time points after silica exposure compared to air control (Fig. 3A,G,M). Also, TPP1 was significantly decreased at 6 and 12 wk after silica inhalation exposure (Fig. 3H,N). Because Tin2 is a central shelterin component which bridges POT1-TPP1 heterodimer and TRF2-TRF1, its inactivation can lead to significant telomere instability, alteration of the shelterin complex, and activation of DNA damage signaling^58^. Reduced levels of telomerase recruitment have been observed after depletion of Tin2, suggesting it to be a regulator of telomerase recruitment at the telomeres^58^. Interestingly, dysregulation of shelterin was initiated at the early time point (3 wk) after silica exposure as was observed by inactivation of Tin2 (Fig. 3C). Both TRFs play an important role in DNA remodeling activities, such as T-Loop formation, however, the exact role of these TRFs in the structural formation of T-Loop is not known. In assessing the influence of silica inhalation on TRF1 and TRF2 levels in rat lung tissue, we observed significantly decreased levels of TRF1 and TRF2 at 6 and 12 wk after silica inhalation compared to air control (Fig. 3J,K) and (Fig. 3P,Q). Notably, we did not observe any changes in TRF1 and TRF2 after 3 wk of silica exposure (Fig. 3D,E). Furthermore, Rap1, which is bound with TRF2 and prevents the localization of homology-directed repair molecules (e.g., PARP1 and SLX4), was significantly downregulated after 12 wk of silica inhalation exposure only (Fig. 3R). No change in Rap1 was observed after 3 and 6 wk of silica exposure (Fig. 3F,L). Collectively, these results suggested that dissociation of shelterin complex was initiated at the early time point (3 wk) after silica inhalation as observed by inactivation of Tin2 and POT1, which aided in keeping double and single stranded telomeric DNA intact.

## Discussion

Telomere integrity is maintained by a complex of shelterin proteins and telomerase activity by adding TTAGGG repeats at the ends of chromosomes, allowing genomic stability that is imperative for an individual’s health and future generations^59^. Alterations in genomic stability could result in the development of cancer and other life threatening pathologies^2,15,17,45^. The six shelterin subunits form a single shelterin complex along with a set of non-shelterin proteins at the ends of chromosomes that all play a dynamic role in telomere maintenance^15^.

The current study further corroborates the findings of our previous study in which we identified telomere instability as observed by significant increases in telomere length and TERT expression at 4 and 32 wk after 15 mg/m^3^ of silica × 6 h/d × 5 d exposure^55^. Here, we provide the first experimental evidence that DNA damage-induced shelterin dysfunction after silica exposure could allow for the development of pulmonary fibrosis. Evidence has indicated that occupational exposure to multiwall carbon nanotubes (MWCNTs) may stimulate EMT and activate myofibroblasts both *in vivo* and *in vitro* leading to fibrosis^60^. Previously, it was reported that silica exposure of 15 mg/m^3^ × 6 h/d × 5 d for 3, 6 and 12 wk exposure resulted in significant lung injury and inflammation in rats. Major histological changes such as type II pneumocyte hyperplasia and evidence of lung fibrosis were observed after 12 wk of silica exposure^56^. In the current study, we observed an increased expression of EMT marker, α-SMA, in paraffin-embedded lung tissue at 12 wk as compared to 3 and 6 wk of silica exposure. DNA damage has been shown to induce the development of pulmonary fibrosis through telomere dysregulation in mice^45^. Interestingly, the presence of DNA damage, as evidenced by an increase in γH2AX-positive cells, was much higher in the 12-wk silica-exposed lung section compared to the 3 and 6 wk groups. Some γH2AX-positive staining, however, was observed in lung tissue sections of 6 and 12 wk of air controls which was consistent with other studies, suggesting that aging is also associated with increased risk of pulmonary fibrosis^2,7^.

Importantly, inactivation of shelterin components has been observed to stimulate the formation of dysfunctional telomeres, leading to cancer initiation and progression^30,49,61^. POT1 interacts with TPP1 located at the single-stranded TTAGGG region of the telomere, and inhibition or inactivation of POT1 could result in the reduction of the telomeric region^33^. Ablation of POT1 can result in the accumulation of increased DDR, telomere dysfunction and fusions in human cells^31,32,34^. Our data demonstrated that development (at 3 wk) and progression (at 6 and 12 wk) of expression of γH2AX, and α-SMA could be responsible for POT1 inactivation after silica inhalation. POT1 and TPP1 interaction protect the 3′ telomere from being recognized as DNA damage^62^. As evidenced by downregulation of TPP1 at 6 and 12 wk of silica exposure, we postulated that TPP1 interaction with POT1 was crucial for maintaining the integrity of 3′ TTAGGG. Tin2 interacts with TPP1 and TRF2 (TRF2-Tin2-TPP1) and maintains telomere length by independently recruiting telomerase^58^. However, the action of telomerase on telomeres is TPP1-dependent and depletion of TIN2 would result in reduced levels of TPP1-mediated telomerase recruitment^38^. Therefore, our data suggested that disruption of Tin2 which was initiated at 3 wk of silica exposure affected TRF2 and TPPI-POT1 interaction on the telomere. Depletion of TRF1 and TRF2 has been reported in the development and progression of DNA damage and pulmonary fibrosis and lung cancer^45,57,63^.

In our previous study^55^, we observed telomere elongation after silica inhalation which could be associated with TRF1 inactivation, because it negatively regulates telomere length. TRF2, one of the subunit of the shelterin complex, prevents chromosomal end-to-end fusions by repressing NHEJ and HDR. However, Rap1, which unites with TRF2, is not involved in NHEJ, and, therefore, its ablation does not prevent chromosomal end fusion^49^. TRF1 and TRF2 gene expression was significantly decreased in lung tissue at 6 and 12 wk of silica exposure; however, no change was observed at 3 wk and in air controls. RTEL1 promotes telomere replication by dismantling the t-loop structure of telomere and its inhibition is associated with telomere dysregulation. Unwinding of the t-loop takes place in S phase through recruitment of RTEL1 by TRF2^42,64^. Here, we observed inactivation of TRF2 and RTEL1, which suggests minimal recruitment of RTEL1 at the telomere.

**Figure 4:**
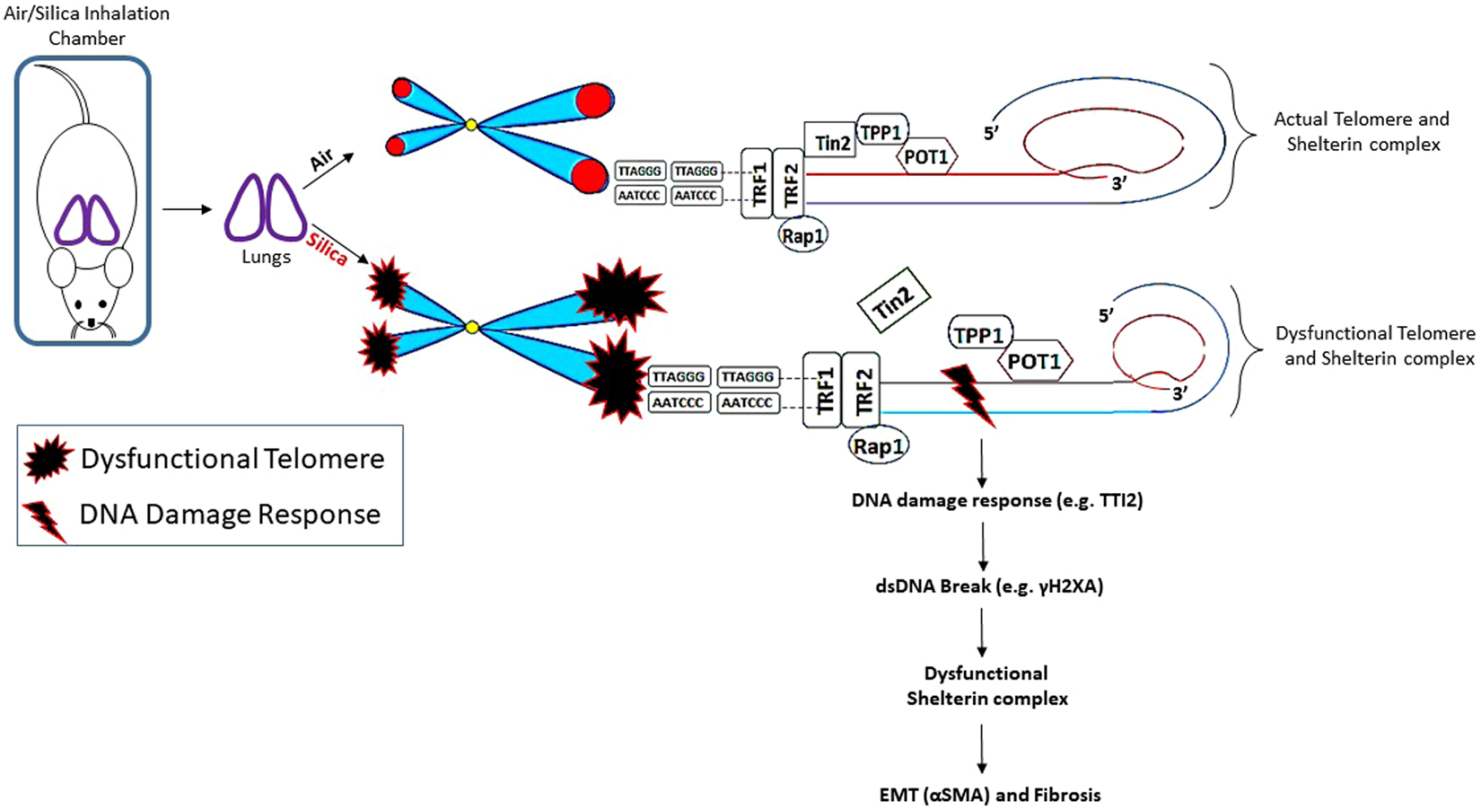
Schematic diagram of the initiation of pulmonary fibrosis after silica inhalation is associated with shelterin dysregulation and dsDNA break in rat lung that may lead to a persistent and severe DDR (TTI2) at telomeres characterized by increased γH2AX and αSMA. Silica inhalation caused an increased staining in the number of γH2AX positive cells and an elevated αSMA expression in lung tissue compared to air control. Initiation of shelterin dysregulation was started after 3 wk of silica exposure as shown by the disruption of Tin2-TPP1 and remained persistent at 6 and 12 wk of silica exposure.

These findings support a model for silica-induced pulmonary fibrosis which involves chronic lung inflammation, persistent DNA damage, a dysfunctional shelterin complex, and the eventual development of pulmonary fibrosis (Fig. 4). We evaluated the effect of silica inhalation exposure for 3, 6 and 12 wk on the shelterin complex (e.g., POT1, TPP1, Tin2, TRF1, TRF2, and Rap1) on rat lung tissues. The potential disruption of the TIN2-TPP1-POT1 complex at the early stage of silica inhalation (3 wk) effectively led to the dysregulation of the shelterin complex at the later time points (6 and 12 wk), which may directly affect DNA protection. In addition, we observed that inactivation of TRF2 could induce DNA damage, as evidenced by an increase in λH2AX-positive cells in lung tissue sections after silica exposure. We also showed that increased expression of λH2AX and α-SMA induced by silica inhalation resulted in shelterin dysfunction that could be a possible mechanism leading to pulmonary fibrosis development.

## Materials and Methods

### Animals

Banked lung samples collected from silica-exposed animals from a previously published study were used to perform the experiments^56^. In this study, 2–3 month old male Fischer 344 rats were obtained from Charles River Laboratories, Wilmington, MA, USA. All the rats were acclimated for approximately 10 days upon receipt, and were housed two rats per cage under controlled room temperature (22–25 °C) and humidity (40–60%). Irradiated Teklad 2918 diet, ad libitum tap water and HEPA-filtered air were provided. During this study all animal procedures used were reviewed and approved by the National Institute for Occupational Safety and Health’s Institutional Animal Care and Use Committee. The animal facilities are specific pathogen-free, environmentally-controlled, and accredited by the AAALAC International (Frederick, MD, USA). All methods in this study were performed in accordance with the relevant guidelines and regulations by the National Institute for Occupational Safety and Health.

### Inhalation exposure of rats to silica aerosol

The silica aerosol generation containing respirable silica particles recommended for rats with mass median diameter of 1.6 µm was obtained from U.S. Silica, Berkeley Springs; WV, USA. Rats were exposed to whole body crystalline silica inhalation system at a concentration of 15 mg/m^3^, 6 hr/d, 5 d/wk for 3, 6, or 12 wk, and silica concentration (15 ± 1 mg/m^3^) were monitored and maintained within the exposure chamber. Control animals were exposed to filtered air. Rats in all groups were euthanized approximately 16 hr following the conclusion of the 3, 6, and 12 wk exposures to air or silica using an intraperitoneal injection of 100–300 mg sodium pentobarbital/kg body weight (Fort Dodge Animal Health; Fort Dodge, IA, USA) followed by exsanguination.

### RNA isolation from lung samples

Lung RNA was isolated and reverse transcribed to cDNA following the conclusion of the 3, 6, and 12 wk exposures to air or silica. RNeasy Fibrous Tissue Mini Kit (Qiagen Inc.; Valencia, CA, USA) was used according to kit instructions. Briefly, 25–30 mg of lung tissue were used to isolate total RNA from the right apical lobe of the control and silica-exposed lung, homogenized in buffer RLT and two 2.4 mm Zirconia beads (BioSpec Products Inc.; Bartlesville, OK, USA) using a mini beadbeater-8 (BioSpec Products Inc.) for 20 sec. The tissue homogenate was centrifuged at 10,000 × g for 10 min at room temperature, and the RNA present in the supernatant was extracted and purified using RNeasy columns.

Following RNA quantification, samples were reverse transcribed using random hexamers (Applied Biosystems, Foster City, CA, USA) and Superscript III (Invitrogen; Carlsbad, CA, USA). Gene expression was analyzed using transcribed cDNA, hypoxanthine-guanine phosphoribosyltranferase (HPRT) was used as the endogenous control.

### Shelterin components, RTEL1 and TTI2 Analysis

Relative mRNA levels of shelterin components along with RTEL1 and TTI2 were determined by quantitative PCR (qPCR). Gene expression was determined using the StepOne Plus (Applied Biosystems, Carlsbad, CA, USA) with pre-designed Assays-on-Demand TaqMan probes and primers including POT1 (Rn01747967_m1), TPP1 (Rn00580350_m1), Tin2 (Rn01481999_g1), TRF1 (Rn01749291_m1), TRF2 (Rn01432601_m1), Rap1 (Rn01762131_m1), TTI2 (Rn01756964_m1), and RTEL1 (Rn01220420_m1) (Thermo Fisher Scientific, Waltham, MA, USA). Using 96-well plates, cDNA was used for gene expression. HPRT was used as the endogenous control.

### Immunofluorescence and Immunohistochemistry for αSMA and γH2AX in the Lungs

The paraffin-embedded sections were kept at 60°C for 1 hr, deparaffinized in xylene, rehydrated by passing through 100%, 95%, and 70% ethanol, and washed 1X in PBS for 5 min. Sections were submerged in 500 ml of antigen retrieval citrate buffer and steam-heated in a standard steamer for 10 min. After the antigen retrieval, endogenous peroxidase was blocked with 3% H_2_O_2_, the sections were rinsed in PBS twice and incubated in blocking solution (2% BSA, 0.1% Triton X-100) for 2–3 hr at room temperature. The sections were incubated with primary antibodies against αSMA (ab5694, dilution 1:200) and γH2AX (ab26350, dilution 1:200) overnight and washed with PBS (3 × 5 min each). One set of the sections were incubated in respective Alexa Fluor-488 or Alexa Fluor-594 secondary antibodies (dilution 1:300) for 1 hr at room temperature followed by washing with PBS (3 × 10 min each). The other set of sections were incubated in HRP-conjugated secondary antibody, and the peroxidase reaction was visualized using diaminobenzidine (DAB). The immunofluorescence sections were mounted using ProLong Gold (Molecular Probes, Eugene, OR). Expression of αSMA and γH2AX positive cells were visualized in the lungs airspace. Images were taken with an Olympus AX70 upright microscope using a 40x objective as previously described^61^.

### Statistical analysis

Statistical differences between the silica and air groups within a time point were compared using a one-way ANOVA. Post-hoc comparisons were made with the Fisher’s least significant difference test. Values represent means ± standard error. Criterion of significance was set at p < 0.05.
